# Fundic Gallbladder Diverticulum Lesion in a Child Suspected to Be Congenital: A Case Report and Scoping Review

**DOI:** 10.3390/pediatric18050117

**Published:** 2026-09-04

**Authors:** Samentha Menager, Piero Farruggia, Salvatore Calderaro, Domenico Bivona, Giovanni Francesco Saia, Marco Guida, Antonino Levita, Chiara Marino, Federica Mescolo, Tiziana Guida Rutilio, Michelangelo Giovanni Sciortino, Barbara Torrente, Flavia Volpe, Giovanni Corsello

**Affiliations:** 1 Unit of Pediatrics, S. Cimino Hospital, Termini Imerese (Palermo), Azienda Sanitaria Provinciale (ASP) Palermo, 90141 Palermo, Italy; samentha.menager@asppalermo.org (S.M.); giovannifrancesco.saia@asppalermo.org (G.F.S.); marco.guida@asppalermo.org (M.G.); chiara.marino@asppalermo.org (C.M.); federica.mescolo@asppalermo.org (F.M.); tiziana.guidarutilio@asppalermo.org (T.G.R.); michelangelogiovanni.sciortino@asppalermo.org (M.G.S.); barbara.torrente@asppalermo.org (B.T.); flavia.volpe@asppalermo.org (F.V.); 2 Unit of Radiology, S. Cimino Hospital, Termini Imerese (Palermo), Azienda Sanitaria Provinciale (ASP) Palermo, 90141 Palermo, Italy; salvatore.calderaro@asppalermo.org (S.C.); domenico.bivona@asppalermo.org (D.B.); 3 Department of Health Promotion, Mother and Child Care, Internal Medicine and Medical Specialties “G. D’Alessandro”, University of Palermo, 90133 Palermo, Italy; giovanni.corsello@unipa.it; 4 Azienda Sanitaria Provinciale (ASP) Palermo, 90141 Palermo, Italy; antonino.levita@asppalermo.org

**Keywords:** gallbladder diverticulum, congenital anomaly, child, ultrasound, scoping review

## Abstract

Gallbladder diverticulum is an exceptionally rare congenital anomaly in children, with only isolated cases reported in the literature. We describe a 10-year-old girl with epilepsy and constipation who presented twice within 15 days with diffuse abdominal pain. Physical examination revealed generalized abdominal tenderness, and laboratory tests were unremarkable. Abdominal ultrasonography showed mild hepatic steatosis and a small fundal gallbladder outpouching measuring approximately 7 mm × 9 mm, suggestive of a diverticulum. Computed tomography (CT) also suggested a small fundal gallbladder diverticulum-like lesion. Notably, the family reported that the patient’s older sister had undergone cholecystectomy for gallstones, during which a gallbladder diverticulum-like lesion was identified; however, because the event had occurred approximately 15 years earlier, only the first abdominal ultrasound report was available for review, and no histopathological reports could be retrieved. The scoping review systematically mapped the available evidence and confirmed that published pediatric experience remains extremely limited. Only two previously published pediatric cases were identified. Given the absence of complications for our patient, conservative management with gastroenterology follow-up was adopted. This case highlights the rarity of congenital gallbladder diverticulum in childhood, the diagnostic challenge of distinguishing true diverticula from anatomical variants, and the importance of correlating imaging findings with the clinical presentation.

## 1. Introduction

Embryologically, the gallbladder develops from the hepatic diverticulum between the 18th and 25th days of gestation. Disturbances during this period may lead to congenital anomalies of the biliary tract, including gallbladder diverticula [1,2]. Diverticula of the gallbladder body and neck may arise from persistent cystohepatic ducts, which connect the developing gallbladder to the liver during embryogenesis. In contrast, fundal diverticula are thought to originate from incomplete vacuolization of the solid gallbladder during embryonic development [1,3].

Gallbladder diverticula can be congenital (true) or acquired (false or pseudo-diverticula); the latter lacks a complete wall and usually results from gallstone-related pressure and inflammation [4,5]. A true gallbladder diverticulum is an exceptionally rare congenital abnormality characterized by a focal outpouching of the gallbladder wall. It is typically solitary, whereas false diverticula tend to be multiple [5,6,7]. It accounts for only 0.06% of all congenital gallbladder anomalies [8] and is exceptionally rare in children; to our knowledge, the available literature consists of only 2 isolated case reports [9,10].

Many reports have highlighted the diagnostic challenges of distinguishing a true diverticulum from other gallbladder anatomical variants. In particular, imaging findings may overlap with those of a Phrygian cap deformity or other benign structural abnormalities, making accurate diagnosis difficult without advanced imaging or surgical confirmation [3,6,11,12].

We report the case of a child with a suspected congenital fundic gallbladder diverticulum-like lesion, identified during evaluation for abdominal pain; to our knowledge, this could be the third pediatric case reported related to gallbladder diverticulum. To better contextualize this exceptionally rare condition, we also conducted a scoping review of the available literature, summarizing the evidence and providing practical considerations for diagnosis and management.

## 2. Methods

A scoping review was conducted to map the available evidence on gallbladder diverticulum, with particular emphasis on pediatric cases, clinical presentation, diagnostic imaging, management, and outcomes. The review was conducted and reported in accordance with the Preferred Reporting Items for Systematic Reviews and Meta-Analyses extension for Scoping Reviews (PRISMA-ScR) guidelines. No review protocol was prospectively registered.

A comprehensive literature search was conducted on 31 May 2026 across PubMed/MEDLINE, Scopus, Web of Science, Embase, and the Cochrane Library. The search strategy combined controlled vocabulary (Medical Subject Headings [MeSH], when applicable) and free-text terms for gallbladder diverticulum. The PubMed search strategy was as follows: (“Gallbladder”[Mesh] OR gallbladder) AND (diverticulum OR diverticula) AND (child OR pediatric OR paediatric). Equivalent search strategies were adapted for the other databases. No language restrictions were applied. Reference lists of all eligible studies and relevant review articles were manually screened to identify additional publications.

Studies reporting congenital or acquired gallbladder diverticulum in children or adults were considered eligible if they provided clinical, diagnostic, imaging, therapeutic, or follow-up information relevant to this review’s objectives. Eligible publications included case reports, case series, observational studies, retrospective analyses, literature reviews, systematic reviews, clinical guidelines, and scoping or narrative reviews.

Two reviewers (G.F.S. and P.F.) independently charted the data using a standardized extraction form developed by the investigators. The form and process were piloted on a small subset of records to ensure consistent interpretation of fields such as diverticulum location, imaging findings, and management. Any ambiguities encountered during piloting were addressed through discussion, resulting in a refined form before full data collection. For study selection, titles, abstracts, and full texts were independently reviewed by the same two reviewers, with disagreements resolved through discussion and consensus. During data extraction, any discrepancies between reviewers were discussed until agreement was reached. If consensus was not possible, a third senior author was consulted.

Data were independently charted using a standardized data extraction form developed by the investigators. The following information was extracted whenever available: year of publication, patient age, sex, diverticulum location, clinical presentation, imaging findings, associated gallbladder abnormalities, treatment strategy, and clinical outcome.

Consistent with scoping review methodology, no formal risk-of-bias or methodological quality assessment of the included studies was performed. The extracted evidence was synthesized descriptively, with particular emphasis on epidemiology, diagnosis, differential diagnosis, management strategies, and clinical outcomes.

However, given its rarity, the search strategy has been overly restrictive, and the small number of pediatric cases identified is a limitation.

**Figure 1:**
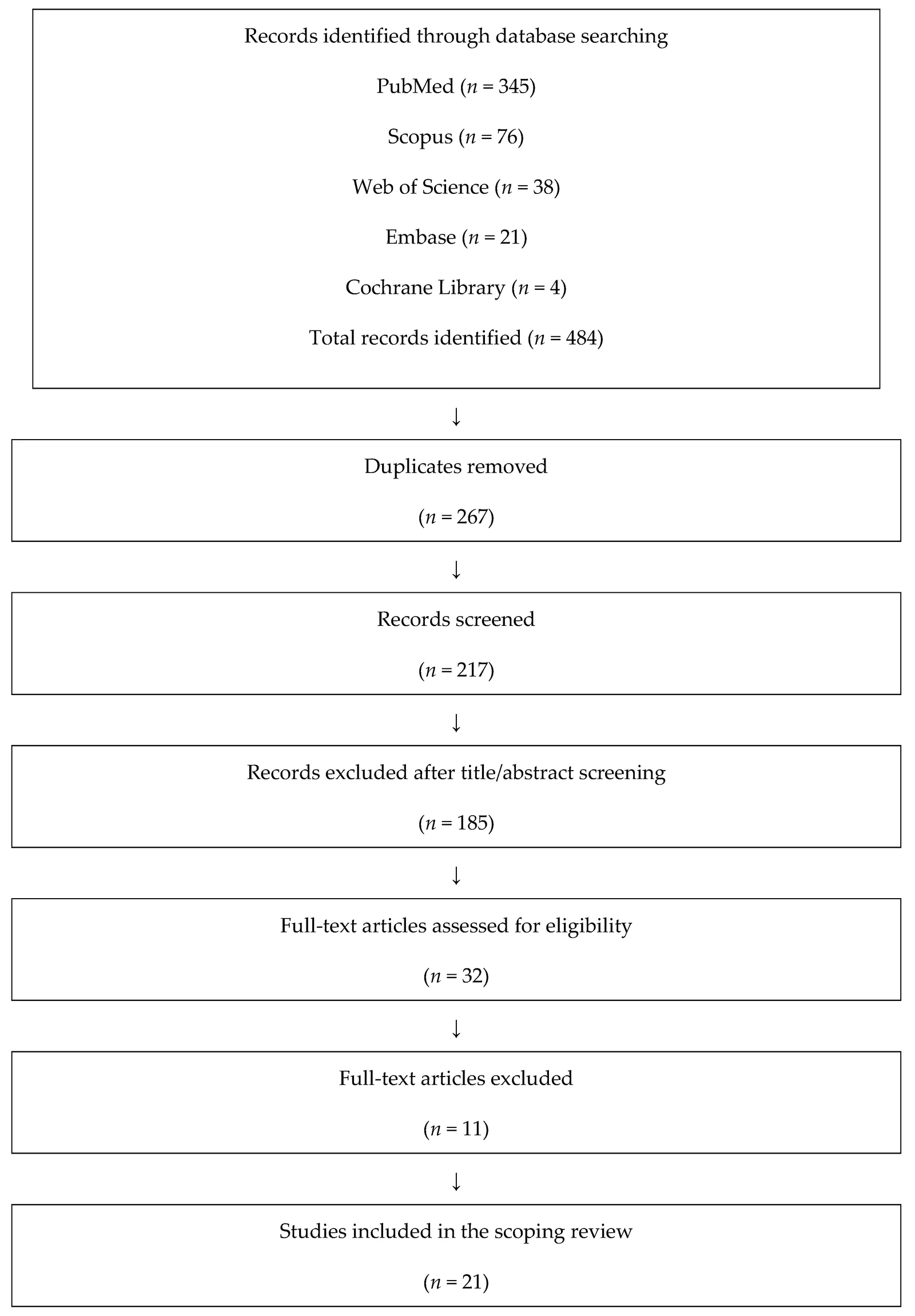
PRISMA flow diagram summarizing the study selection process for the scoping review.

The study selection process is summarized in the PRISMA flow diagram (Figure 1). The completed PRISMA-ScR checklist is included as Supplementary Materials (Table S2).

### Results of the Scoping Review

The literature search identified 484 records, of which 267 were duplicates and removed. After title and abstract screening, 32 full-text articles were assessed for eligibility, and 21 studies were ultimately included in the scoping review. The complete study selection process is illustrated in Figure 1. Most available evidence consisted of isolated case reports or small case series. No prospective studies or randomized trials were identified. Most publications involved adult patients, whereas only 2 pediatric cases had been reported before the present report. The included studies primarily addressed the embryological origin of gallbladder diverticula, imaging characteristics, differential diagnosis, clinical presentation, and therapeutic management. Overall, the available evidence was scarce and predominantly low-level, consisting mainly of isolated case reports and small case series. A summary of the characteristics of the 2 published pediatric cases and the present patient is provided in Supplementary Table S1.

## 3. Case Presentation

A 10-year-old girl with a history of epilepsy and chronic constipation presented to the emergency department for the second time within 15 days due to diffuse abdominal pain. Her family history was notable for an older sister who had undergone cholecystectomy for gallstones, during which a gallbladder diverticulum- like lesion had been identified. Unfortunately, because the event had occurred approximately 15 years earlier, only the first abdominal ultrasound report was available for review, but no histopathological reports could be retrieved, as neither the family nor the treating hospital had retained them.

On physical examination, the patient appeared in good general condition. The examination was unremarkable except for diffuse abdominal tenderness on palpation. Laboratory investigations, including complete blood count, liver function tests GOT 32 U/L (5–31), GPT 23 U/L (5–34), pancreatic enzymes, renal function tests, serum electrolytes, blood glucose, C-reactive protein, and urinalysis, were all within normal limits.

**Figure 2:**
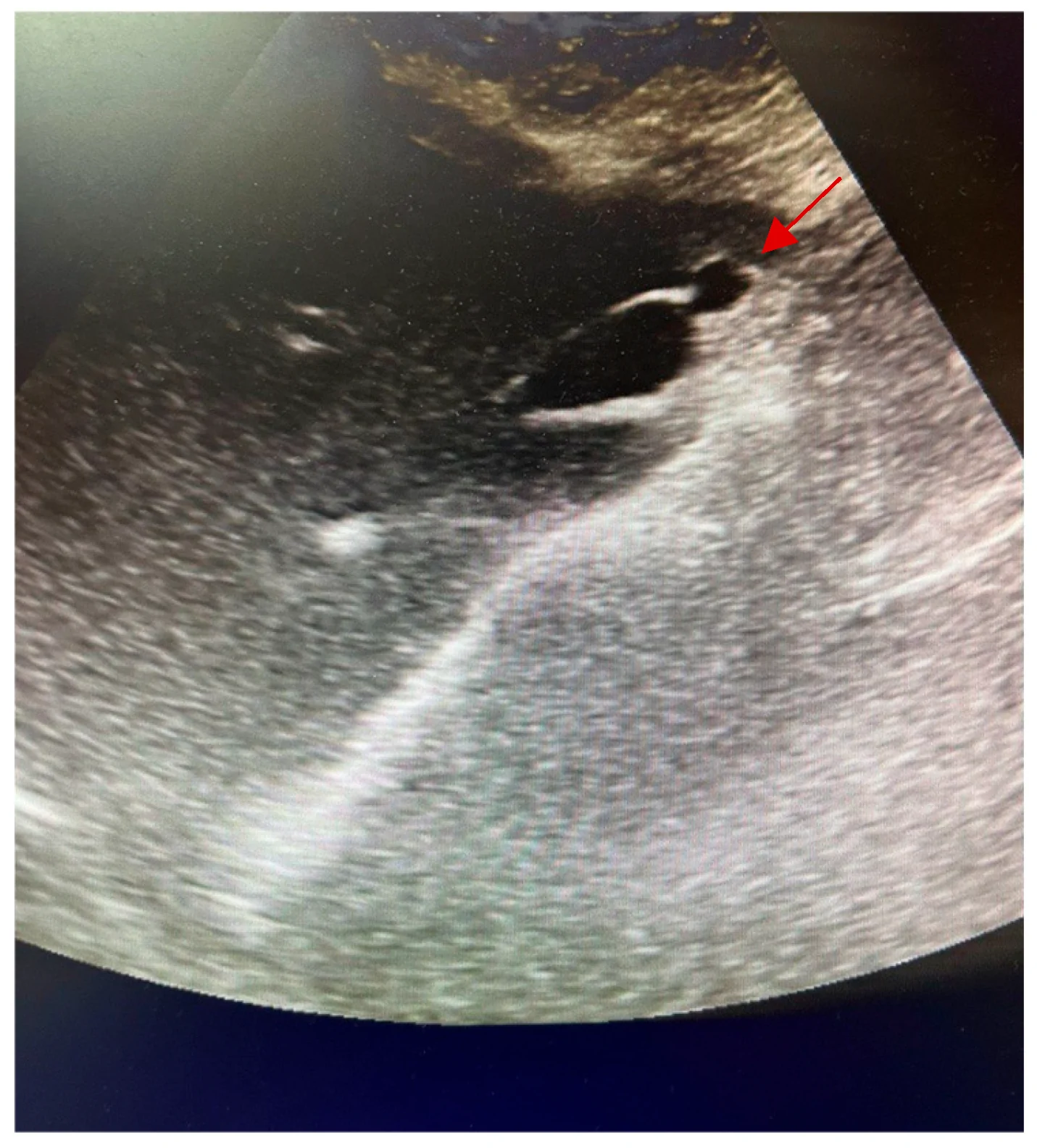
Abdominal ultrasound showing a small cystic outpouching arising from the gallbladder’s fundus (red arrow).

**Figure 3:**
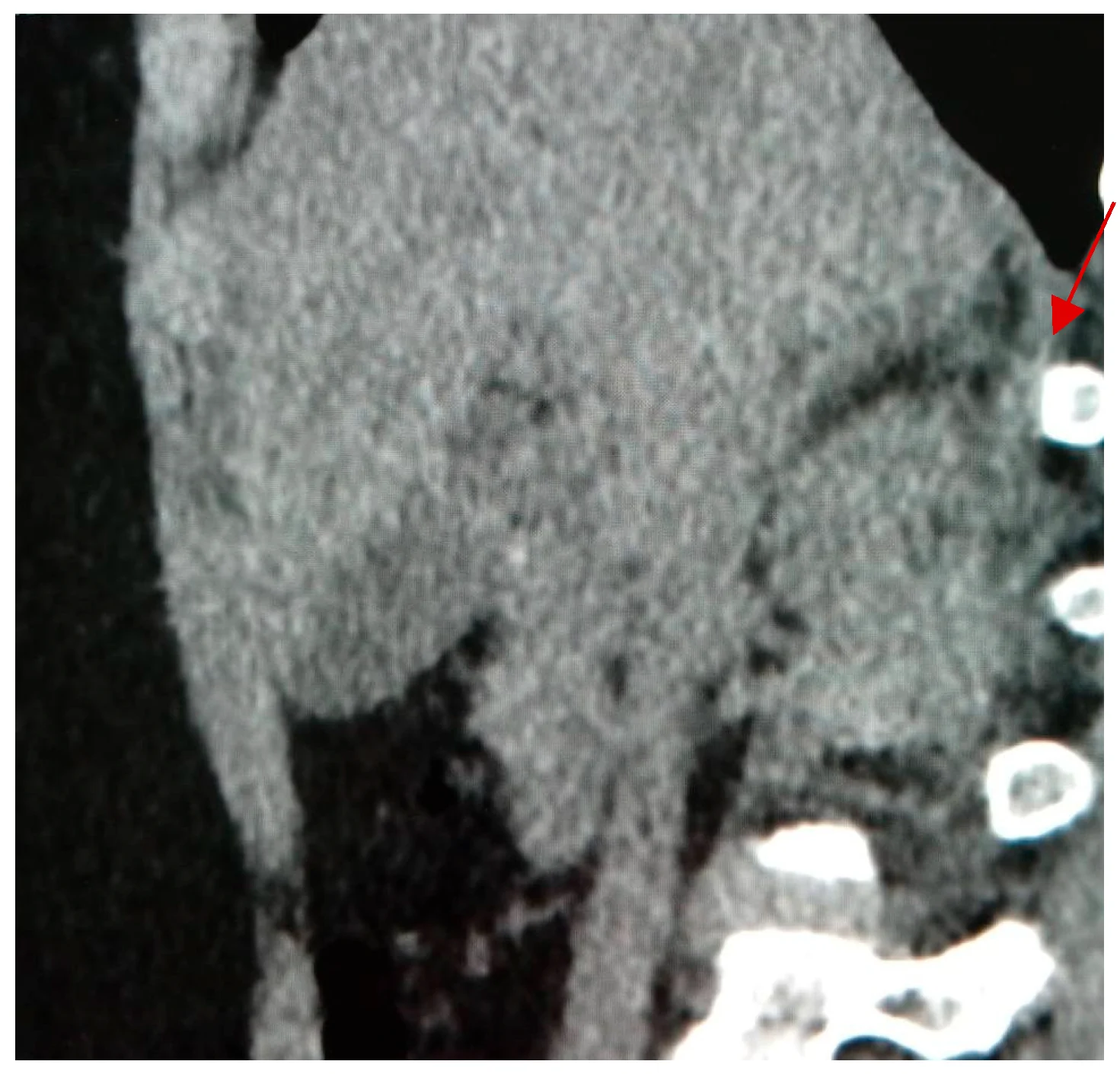
CT image showing a small fundal outpouching of the gallbladder (red arrow), highly suggestive of a gallbladder diverticulum.

Constipation was considered the most likely cause of the symptoms, and an evacuative enema was administered. However, because abdominal pain persisted, an abdominal ultrasound was performed. Ultrasonography demonstrated mild hepatic steatosis and a fundic gallbladder diverticulum-like structure, appearing as a small, cystic outpouching measuring approximately 7 mm × 9 mm that communicates with the gallbladder lumen (Figure 2 and Figure 3). No additional abnormalities were identified.

Because the symptoms persisted, an abdominal CT scan was obtained 24 h later. The examination confirmed mild hepatic steatosis and a small diverticular-like outpouching of the gallbladder wall. A second expert radiologist, blinded to the initial interpretation, independently reviewed both the ultrasound and CT images and supported the diagnosis of a probable gallbladder diverticulum. On this basis, magnetic resonance cholangiopancreatography (MRCP), which has been reported to be useful for diagnosis in some studies [2,3], was not performed. Because the patient’s symptoms progressively resolved and a definitive causal link between the imaging finding and her symptoms could not be established, conservative management was adopted. No additional abdominal pain occurred during 15 months of follow-up.

## 4. Discussion

Congenital gallbladder diverticulum is a rare malformation, often discovered incidentally on imaging for abdominal symptoms. It is extremely uncommon in children; this review included only two previously documented pediatric cases. The first involved a 3-year-old boy with cholecystitis and cholelithiasis arising from a congenital diverticulum, treated surgically. The second was a 15-year-old boy with gallbladder diverticulum identified in the setting of chronic calculous cholecystitis. Both presented with biliary symptoms and underwent cholecystectomy, with no reported postoperative complications (see Supplementary Table S1). As a result, the clinical significance and optimal treatment approaches for this condition remain poorly understood.

Ultrasonography is the first-line imaging modality and may show a cystic outpouching communicating with the gallbladder lumen. The main differential diagnoses (Table 1) include a Phrygian cap, Hartmann’s pouch, adenomyomatosis, gallbladder duplication [13], and choledochal cyst variants [14]. In our patient, the narrow neck connecting the outpouching directly to the gallbladder fundus, without evidence of a separate biliary connection or ductal communication, supported the diagnosis of a gallbladder diverticulum [1,4,11,15,16,17].

Approximately half of gallbladder diverticula arise from the fundus, with the remainder located in the body or neck. Reported manifestations range from asymptomatic lesions to abdominal pain, nausea, vomiting, cholecystitis, cholelithiasis, and recurrent cholangitis [1,3,5,7,8,9,10,14,15]. In our patient, chronic constipation may have accounted for the abdominal pain, and a definitive causal relationship between the symptoms and the diverticulum cannot be established [1,12,18].

The possible presence of a similar lesion in the patient’s older sister is noteworthy. Although familial clustering has been reported for other gastrointestinal diverticular disorders, hereditary predisposition to gallbladder diverticulum has not been established, and familial occurrence has not been previously described. This observation may therefore be coincidental, and the lack of histopathological reports and further documentation remains a major limitation [19,20].

Management should be individualized. Conservative follow-up is reasonable for asymptomatic or minimally symptomatic patients, whereas surgery may be considered for persistent symptoms, gallstones, complications, or substantial diagnostic uncertainty. Given the favorable course and absence of complications, conservative management was appropriate in this case. The principal limitation is the lack of surgical and histopathological confirmation; however, concordant assessments by two experienced radiologists and characteristic ultrasound and CT findings strongly support the diagnosis [4,12,14,21,22,23].

## 5. Conclusions

Congenital gallbladder diverticulum is extremely rare in children, and this case adds to the limited pediatric literature. The imaging and clinical findings suggest that awareness of this anomaly could help clinicians include it in the differential diagnosis of unusual gallbladder abnormalities and encourage more careful interpretation of imaging results, potentially preventing unnecessary surgery. However, as this is a single case, it does not by itself alter diagnosis or management practices; instead, it underscores the clinical significance and the need for more pediatric cases to better understand diagnosis and treatment options.

## 6. Future Directions

Additional pediatric cases are needed to clarify the natural history, clinical significance, and optimal management of congenital gallbladder diverticulum. Given the extreme rarity of this anomaly in children, establishing multicenter registries or collaborative case collections would enable more accurate characterization of its epidemiology, clinical spectrum, imaging features, and long-term outcomes.

The possible familial occurrence observed in our report also raises the question of a familial predisposition. Although this association may be coincidental, future studies should systematically assess family history and explore potential genetic or embryological mechanisms underlying the development of this anomaly.

Further research is needed to validate imaging criteria that reliably distinguish true congenital diverticula from anatomical variants, such as the Phrygian cap or other gallbladder abnormalities, thereby reducing diagnostic uncertainty and avoiding unnecessary surgical intervention. Finally, prospective follow-up studies are warranted to clarify the risk of long-term complications, including gallstone formation, recurrent biliary symptoms, inflammation, and the need for surgical treatment, and to enable the development of evidence-based recommendations for surveillance and management.

## Figures and Tables

**Table 1:** Differential diagnosis with other cystic lesions adjacent to the gallbladder.

Lesions	Imaging Characteristics	Main Clinical Characteristics
Gallbladder duplication	Usually has two separate lumina, often with distinct cystic ducts, and is best characterized by MRCP to delineate biliary anatomy.	Asymptomatic, Incidental finding.
Choledochal cyst	Typically communicates with the biliary tree rather than the gallbladder and may show intrahepatic ductal dilatation.	Abdominal pain, jaundice, palpable mass.
Cystic duct diverticulum	Arises from the cystic duct itself and requires cholangiographic imaging to confirm ductal continuity.	Nonspecific right upper quadrant pain, jaundice, or acute pancreatitis.
Other pericholecystic cystic lesions (Focal adenomyosis, pericholecystic fluid, ciliated foregut cysts)	Generally, lack direct communication with the gallbladder lumen and may have distinct wall characteristics on imaging.	Asymptomatic, Incidental finding.
Phrygian cap	Folding of the gallbladder fundus that creates an apparent septation but lacks a narrow neck communicating with the lumen.	Asymptomatic, Incidental finding.
Hartmann’s pouch	Posteromedial outpouching of the gallbladder neck; it is located at the neck/cystic duct region.	Associated with gallstones and chronic cholecystitis.
Adenomyomatosis	Characterized by mural thickening with intramural Rokitansky–Aschoff sinuses that may appear as small cystic spaces within a thickened wall.	Often asymptomatic but can cause right upper quadrant pain, biliary colic, nausea, or dyspepsia.

## Data Availability

The original contributions presented in this study are included in the article. Further inquiries can be directed to the corresponding author.

## Supplementary Materials

The following supporting information can be downloaded at: https://www.mdpi.com/article/10.3390/pediatric18050117/s1, Table S1: Published pediatric cases of congenital gallbladder diverticulum identified through the scoping review [9,10]; Table S2: Preferred Reporting Items for Systematic reviews and Meta-Analyses extension for Scoping Reviews (PRISMA-ScR) Checklist.

## Abbreviations

The following abbreviations are used in this manuscript:

CT	Computed Tomography
US	Ultrasonography
MeSH	Medical Subject Headings
MRCP	Magnetic Resonance Cholangiopancreatography
PRISMA-ScR	Preferred Reporting Items for Systematic Reviews and Meta-Analyses Extension for Scoping Reviews
