# Supplementary material for: Fundic Gallbladder Diverticulum Lesion in a Child Suspected to Be Congenital: A Case Report and Scoping Review
Source: Pediatr Rep. 2026 Sep 4;18(5):117. doi: 10.3390/pediatric18050117 (PMC13600022; doi:10.3390/pediatric18050117)
Supplement: Supplementary file 1 [file pediatrrep-18-00117-s001.zip › Table S1.pdf]

**Supplementary Table S1**

**Published pediatric cases of congenital gallbladder diverticulum identified through the scoping review**

| Study | Patient | Clinical presentation | Diagnostic findings | Treatment | Outcome / Main findings |
|---|---|---|---|---|---|
| Bogatko FH, Mehlman J. (1947) [10] | 3-year-old boy | Acute cholecystitis associated with cholelithiasis | Congenital gallbladder diverticulum diagnosed at surgery | Cholecystectomy | Diverticulum associated with gallstones and chronic inflammation. Uneventful postoperative course. |
| Kramer AJ et al. (1998) [9] | Pediatric patient* | Abdominal symptoms leading to diagnostic evaluation | Gallbladder diverticulum identified by imaging and confirmed surgically | Cholecystectomy | Confirmed the rarity of congenital gallbladder diverticulum in childhood and reviewed the previously published literature. Uneventful postoperative outcome. |
| Present case (Menager et al., 2026) | 10-year-old girl | Recurrent diffuse abdominal pain | Ultrasonography demonstrated a 7 × 9 mm fundal diverticulum communicating with the gallbladder lumen; CT confirmed the diagnosis | Conservative management | Third pediatric case reported. First case suggesting possible familial occurrence, as the patient's older sister previously underwent cholecystectomy with a confirmed gallbladder diverticulum. Complete symptom resolution with no recurrence during 15 months of follow-up. |

**The original article does not report all demographic characteristics in the available description.*


9. Kramer, A.J.; Bregman, A.; Zeddies, C.A.; Guynn, V.L. Gallbladder diverticulum: A case report and review of the literature. Am. Surg. 1998, 64, 298–301.

10. Bogatko, F.H.; Mehlman, J. Cholecystitis and cholelithiasis in a congenital diverticulum of the gall bladder in a three-year-old boy. J. Pediatr. 1947, 31, 75–77. https://doi.org/10.1016/S0022-3476(47)80221-5.
